# RETRACTION: Effects of Dietary Pectin and *Lactobacillus salivarius* ATCC 11741 on Growth Performance, Immunocompetence, Gut Microbiota, Antioxidant Capacity, and Disease Resistance in Narrow‐Clawed Crayfish, *Postantacus leptodactylus*

**DOI:** 10.1155/anu/9891417

**Published:** 2026-07-14

**Authors:** Aquaculture Nutrition

RETRACTION: S. D. S. Jastaniah, H. Hafsan, C.‐J. Tseng, et al., “Effects of Dietary Pectin and *Lactobacillus salivarius* ATCC 11741 on Growth Performance, Immunocompetence, Gut Microbiota, Antioxidant Capacity, and Disease Resistance in Narrow‐Clawed Crayfish, *Postantacus leptodactylus*,” *Aquaculture Nutrition* 2022, no. 1 (2022): 1–13, https://doi.org/10.1155/2022/1861761.

The above article, published online on 2 November 2022 in Wiley Online Library (wileyonlinelibrary.com), has been retracted by agreement between the Journal Editor‐in‐Chief, Antony J. Prabhu Philip; and John Wiley & Sons Ltd.

The retraction has been agreed upon following an investigation into concerns initially raised by a third party over the authorship of the article [1]. Following an investigation into these concerns, inconsistencies have been identified around the contributions of individual authors and the accountability of this work. As a result, we no longer have confidence in the integrity of the article’s content, and it is therefore retracted.

The authors did not respond when they were informed of the decision to retract.
